# 1-(2-Chloro­benzo­yl)-3-(2,3-dimethyl­phen­yl)thio­urea

**DOI:** 10.1107/S1600536812048209

**Published:** 2012-11-30

**Authors:** M. Khawar Rauf, Masahiro Ebihara, Amin Badshah

**Affiliations:** a Department of Chemistry, Quaid-i-Azam University, Islamabad 45320, Pakistan; b Department of Chemistry, Faculty of Engineering, Gifu University Yanagido, Gifu 501-1193, Japan

## Abstract

The dihedral angle between the two phenyl groups in the title compound, C_16_H_15_ClN_2_OS, is 14.88 (4)°. An intra­molecular N—H⋯O hydrogen bond occurs. In the crystal, pairs of N—H⋯S hydrogen bonds link the mol­ecules into centrosymmetric dimers.

## Related literature

For background and a related structure, see: Rauf *et al.* (2012 ▶). For a description of the Cambridge Structural Database, see: Allen *et al.* (2002 ▶).
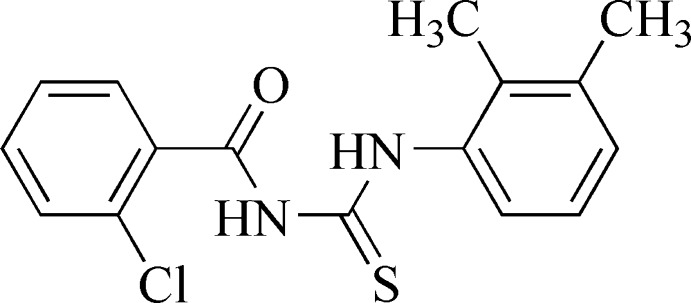


## Experimental

### 

#### Crystal data

C_16_H_15_ClN_2_OS
*M* *_r_* = 318.81
Triclinic, 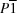

*a* = 7.489 (3) Å
*b* = 9.338 (4) Å
*c* = 13.274 (5) Å
α = 65.674 (13)°
β = 69.975 (16)°
γ = 73.639 (17)°
*V* = 783.9 (5) Å^3^
*Z* = 2
Mo *K*α radiation
μ = 0.38 mm^−1^
*T* = 123 K
0.50 × 0.35 × 0.28 mm

#### Data collection

Rigaku/MSC Mercury CCD diffractometer
6180 measured reflections
3506 independent reflections
3373 reflections with *I* > 2σ(*I*)
*R*_int_ = 0.062

#### Refinement

*R*[*F* ^2^ > 2σ(*F* ^2^)] = 0.036
*wR*(*F* ^2^) = 0.090
*S* = 1.08
3506 reflections
192 parameters
H-atom parameters constrained
Δρ_max_ = 0.32 e Å^−3^
Δρ_min_ = −0.31 e Å^−3^

### 

Data collection: *CrystalClear* (Molecular Structure Corporation & Rigaku, 2001 ▶); cell refinement: *CrystalClear*; data reduction: *CrystalClear*; program(s) used to solve structure: *SIR97* (Altomare *et al.*, 1999 ▶); program(s) used to refine structure: *SHELXL97* (Sheldrick, 2008 ▶); molecular graphics: *ORTEPII* (Johnson, 1976 ▶); software used to prepare material for publication: *Yadokari-XG* (Wakita, 2001 ▶; Kabuto *et al.*, 2009 ▶).

## Supplementary Material

Crystal structure: contains datablock(s) I, global. DOI: 10.1107/S1600536812048209/hg5274sup1.cif

Structure factors: contains datablock(s) I. DOI: 10.1107/S1600536812048209/hg5274Isup2.hkl

Supplementary material file. DOI: 10.1107/S1600536812048209/hg5274Isup3.cml

Additional supplementary materials:  crystallographic information; 3D view; checkCIF report

## Figures and Tables

**Table 1:** Hydrogen-bond geometry (Å, °)

*D*—H⋯*A*	*D*—H	H⋯*A*	*D*⋯*A*	*D*—H⋯*A*
N1—H1⋯S1^i^	0.88	2.46	3.3104 (15)	164
N2—H2⋯O1	0.88	2.00	2.6866 (17)	134

Symmetry code: (i) 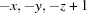.

**Fig. 1.**
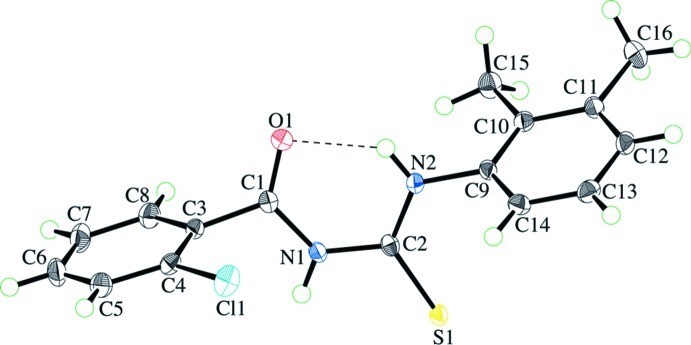
ORTEP of (I). Displacement ellipsoids are drawn at the 50% probability level. Hydrogen bonds shown as dashed lines.

**Fig. 2.**
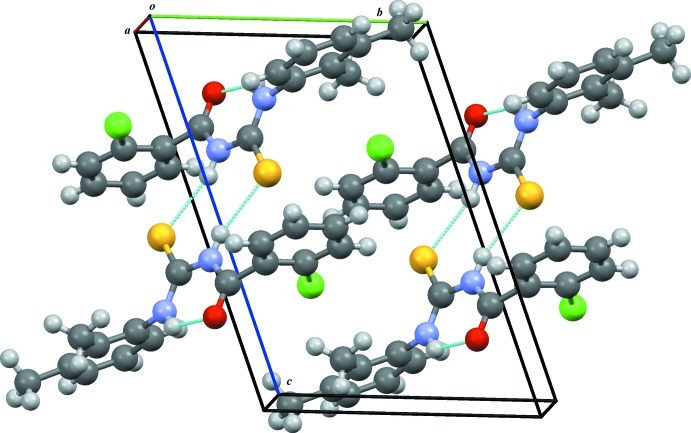
Packing diagram of (I). Hydrogen bonds shown as dashed lines.

## supplementary crystallographic information

### Comment

The background to this study has been set out in our previous work for the structural chemistry of *N*,*N'*-disubstituted thiourea (Rauf *et al.*, 2012) and their coordination chemistry. Herein, as a continuation of these crytallographic studies, the structure of the title compound (I) is described, Fig. 1. Compared to *N*-benzoyl-*N'*-phenylthioureas [Cambridge Structural Database (*Mogul* Version 1.7; Allen, 2002], the methyl substitutions at C(10) and C(11)on phenyl ring, implies no significant effect on these bond lengths. and show the molecule to exist in the thione form with typical thiourea C—S and C—O bonds, as well as shortened C—N bond lengths. The dihedral angles to the O(1) C(1) N(1) C(2) N(2)S(1) plane are 79.89 (2)° for the ring formed by C(3) to C(8) and 65.32 (2)° for the ring formed by C(9) to C(14). An intramolecular N—H···O H–bond is present (Table 1), forming a six-membered ring commonly observed in this class of compounds (Rauf *et al.*, 2012). In the crystal packing of (I), intermolecular N—H···S H–bonds link the molecules into centrosymmetric dimers (Fig.2).

### Experimental

Freshly prepared 2-chlorobenzoylisothiocyanate (1.98 g, 10 mmol) was dissolved in acetone (30 ml) and stirred for 30 minutes. Afterwards neat 2,3-dimethylaniline(1.21 g, 10 mmol) was added and the resulting mixture was stirred for 2 h. The reaction mixture was then poured into acidified water and stirred well. The solid product was separated and washed with deionized water and purified by recrystallization from methanol/ 1,1-dichloromethane (1:1 *v/v*) to give fine crystals of the title compound (I), with an overall yield of 95% (3.02 g). M.P; 180–181°C Anal. calcd. for C_16_ H_15_Cl N_2_ O S; C, 60.28 H, 4.74 N, 8.79 S, 10.06 Found: C, 60.22 H, 4.73 N, 8.78 S, 10.01.

### Refinement

Hydrogen atoms were included in calculated positions and refined as riding on their parent atom with N—H = 0.88 Å and *U*_iso_(H) = 1.2U(N_eq_), C_aromatic_—H = 0.95 Å and *U*_iso_(H) = 1.2U(C_eq_) or C—H = 0.98 Å and *U*_iso_(H) = 1.5U(C_eq_), for methyl C atoms.

### Crystal data

**Table:**

C_16_H_15_ClN_2_OS	*Z* = 2
*M**_r_* = 318.81	*F*(000) = 332
Triclinic, *P*1	*D*_x_ = 1.351 Mg m^−^^3^
Hall symbol: -P 1	Mo *K*α radiation, λ = 0.71070 Å
*a* = 7.489 (3) Å	Cell parameters from 2732 reflections
*b* = 9.338 (4) Å	θ = 3.0–27.5°
*c* = 13.274 (5) Å	µ = 0.38 mm^−^^1^
α = 65.674 (13)°	*T* = 123 K
β = 69.975 (16)°	Prism, colorless
γ = 73.639 (17)°	0.50 × 0.35 × 0.28 mm
*V* = 783.9 (5) Å^3^	

### Data collection

**Table:**

Rigaku/MSC Mercury CCD diffractometer	3373 reflections with *I* > 2σ(*I*)
Radiation source: Rotating Anode	*R*_int_ = 0.062
Graphite Monochromator monochromator	θ_max_ = 27.5°, θ_min_ = 3.5°
Detector resolution: 14.62 pixels mm^-1^	*h* = −6→9
ω scans	*k* = −9→12
6180 measured reflections	*l* = −10→17
3506 independent reflections	

### Refinement

**Table:**

Refinement on *F*^2^	Primary atom site location: structure-invariant direct methods
Least-squares matrix: full	Secondary atom site location: difference Fourier map
*R*[*F*^2^ > 2σ(*F*^2^)] = 0.036	Hydrogen site location: inferred from neighbouring sites
*wR*(*F*^2^) = 0.090	H-atom parameters constrained
*S* = 1.08	*w* = 1/[σ^2^(*F*_o_^2^) + (0.0371*P*)^2^ + 0.4197*P*] where *P* = (*F*_o_^2^ + 2*F*_c_^2^)/3
3506 reflections	(Δ/σ)_max_ < 0.001
192 parameters	Δρ_max_ = 0.32 e Å^−^^3^
0 restraints	Δρ_min_ = −0.31 e Å^−^^3^

### Special details

**Table:**

Geometry. All e.s.d.'s (except the e.s.d. in the dihedral angle between two l.s. planes) are estimated using the full covariance matrix. The cell e.s.d.'s are taken into account individually in the estimation of e.s.d.'s in distances, angles and torsion angles; correlations between e.s.d.'s in cell parameters are only used when they are defined by crystal symmetry. An approximate (isotropic) treatment of cell e.s.d.'s is used for estimating e.s.d.'s involving l.s. planes.
Refinement. Refinement of *F*^2^ against ALL reflections. The weighted *R*-factor *wR* and goodness of fit *S* are based on *F*^2^, conventional *R*-factors *R* are based on *F*, with *F* set to zero for negative *F*^2^. The threshold expression of *F*^2^ > σ(*F*^2^) is used only for calculating *R*-factors(gt) *etc*. and is not relevant to the choice of reflections for refinement. *R*-factors based on *F*^2^ are statistically about twice as large as those based on *F*, and *R*- factors based on ALL data will be even larger.

### Fractional atomic coordinates and isotropic or equivalent isotropic displacement parameters (Å^2^)

**Table:**

	*x*	*y*	*z*	*U*_iso_*/*U*_eq_	
C1	0.38592 (19)	0.07516 (15)	0.27734 (11)	0.0162 (3)	
O1	0.44634 (15)	0.17028 (13)	0.18262 (9)	0.0245 (2)	
N1	0.20232 (16)	0.09731 (13)	0.34511 (9)	0.0170 (2)	
H1	0.1791	0.0275	0.4152	0.020*	
C2	0.04854 (19)	0.21635 (15)	0.31664 (11)	0.0151 (2)	
S1	−0.15915 (5)	0.22091 (4)	0.41821 (3)	0.01758 (10)	
N2	0.07877 (16)	0.31921 (13)	0.20967 (9)	0.0172 (2)	
H2	0.1972	0.3159	0.1655	0.021*	
C3	0.51000 (18)	−0.07725 (15)	0.32976 (11)	0.0157 (3)	
C4	0.48935 (19)	−0.22203 (16)	0.33182 (11)	0.0175 (3)	
C5	0.6119 (2)	−0.36192 (17)	0.37358 (12)	0.0234 (3)	
H5	0.5988	−0.4597	0.3728	0.028*	
C6	0.7535 (2)	−0.35614 (18)	0.41627 (13)	0.0269 (3)	
H6	0.8371	−0.4512	0.4460	0.032*	
C7	0.7751 (2)	−0.21271 (19)	0.41617 (13)	0.0258 (3)	
H7	0.8721	−0.2102	0.4462	0.031*	
C8	0.6538 (2)	−0.07287 (17)	0.37186 (12)	0.0211 (3)	
H8	0.6695	0.0255	0.3704	0.025*	
Cl1	0.30905 (5)	−0.22869 (5)	0.28008 (3)	0.02652 (11)	
C9	−0.07073 (18)	0.43554 (16)	0.16191 (10)	0.0155 (3)	
C10	−0.06255 (19)	0.59756 (16)	0.12305 (11)	0.0166 (3)	
C11	−0.2070 (2)	0.70696 (16)	0.07143 (11)	0.0188 (3)	
C12	−0.3533 (2)	0.65092 (18)	0.06365 (11)	0.0206 (3)	
H12	−0.4519	0.7251	0.0304	0.025*	
C13	−0.3589 (2)	0.48910 (18)	0.10335 (11)	0.0213 (3)	
H13	−0.4604	0.4533	0.0973	0.026*	
C14	−0.2158 (2)	0.38012 (17)	0.15185 (11)	0.0191 (3)	
H14	−0.2166	0.2690	0.1779	0.023*	
C15	0.0930 (2)	0.65647 (18)	0.13585 (13)	0.0236 (3)	
H15C	0.1687	0.5671	0.1833	0.035*	
H15A	0.0342	0.7369	0.1724	0.035*	
H15B	0.1773	0.7038	0.0602	0.035*	
C16	−0.2044 (2)	0.88359 (18)	0.02548 (14)	0.0299 (3)	
H16C	−0.3084	0.9405	−0.0127	0.045*	
H16A	−0.0802	0.9059	−0.0295	0.045*	
H16B	−0.2229	0.9192	0.0888	0.045*	

### Atomic displacement parameters (Å^2^)

**Table:**

	*U*^11^	*U*^22^	*U*^33^	*U*^12^	*U*^13^	*U*^23^
C1	0.0165 (6)	0.0149 (6)	0.0178 (6)	−0.0008 (5)	−0.0061 (5)	−0.0059 (5)
O1	0.0191 (5)	0.0235 (5)	0.0202 (5)	−0.0012 (4)	−0.0032 (4)	−0.0004 (4)
N1	0.0172 (5)	0.0141 (5)	0.0143 (5)	0.0016 (4)	−0.0037 (4)	−0.0030 (4)
C2	0.0173 (6)	0.0126 (5)	0.0161 (6)	−0.0001 (5)	−0.0061 (5)	−0.0059 (5)
S1	0.01736 (17)	0.01515 (16)	0.01454 (16)	0.00274 (11)	−0.00288 (12)	−0.00425 (12)
N2	0.0138 (5)	0.0177 (5)	0.0152 (5)	0.0010 (4)	−0.0040 (4)	−0.0031 (4)
C3	0.0140 (6)	0.0156 (6)	0.0149 (6)	0.0003 (5)	−0.0025 (5)	−0.0055 (5)
C4	0.0149 (6)	0.0193 (6)	0.0183 (6)	−0.0020 (5)	−0.0018 (5)	−0.0092 (5)
C5	0.0230 (7)	0.0174 (6)	0.0253 (7)	0.0014 (5)	−0.0021 (6)	−0.0095 (5)
C6	0.0234 (7)	0.0227 (7)	0.0270 (7)	0.0076 (6)	−0.0087 (6)	−0.0070 (6)
C7	0.0185 (7)	0.0310 (8)	0.0285 (7)	0.0027 (6)	−0.0112 (6)	−0.0111 (6)
C8	0.0191 (6)	0.0216 (7)	0.0245 (7)	−0.0018 (5)	−0.0078 (5)	−0.0094 (5)
Cl1	0.02276 (19)	0.0319 (2)	0.0347 (2)	−0.00257 (14)	−0.00959 (15)	−0.02058 (16)
C9	0.0147 (6)	0.0169 (6)	0.0111 (5)	0.0013 (5)	−0.0037 (4)	−0.0037 (5)
C10	0.0157 (6)	0.0184 (6)	0.0138 (5)	−0.0011 (5)	−0.0029 (5)	−0.0057 (5)
C11	0.0184 (6)	0.0181 (6)	0.0153 (6)	0.0013 (5)	−0.0038 (5)	−0.0046 (5)
C12	0.0162 (6)	0.0260 (7)	0.0146 (6)	0.0025 (5)	−0.0055 (5)	−0.0048 (5)
C13	0.0167 (6)	0.0297 (7)	0.0170 (6)	−0.0045 (5)	−0.0050 (5)	−0.0069 (5)
C14	0.0206 (6)	0.0184 (6)	0.0160 (6)	−0.0037 (5)	−0.0041 (5)	−0.0040 (5)
C15	0.0229 (7)	0.0225 (7)	0.0283 (7)	−0.0031 (5)	−0.0097 (6)	−0.0096 (6)
C16	0.0303 (8)	0.0173 (7)	0.0344 (8)	0.0012 (6)	−0.0100 (7)	−0.0037 (6)

### Geometric parameters (Å, º)

**Table:**

C1—O1	1.2211 (17)	C8—H8	0.9500
C1—N1	1.3743 (17)	C9—C14	1.392 (2)
C1—C3	1.5017 (18)	C9—C10	1.395 (2)
N1—C2	1.3912 (17)	C10—C11	1.4115 (19)
N1—H1	0.8800	C10—C15	1.504 (2)
C2—N2	1.3299 (17)	C11—C12	1.389 (2)
C2—S1	1.6770 (14)	C11—C16	1.508 (2)
N2—C9	1.4385 (17)	C12—C13	1.388 (2)
N2—H2	0.8800	C12—H12	0.9500
C3—C8	1.389 (2)	C13—C14	1.385 (2)
C3—C4	1.3917 (19)	C13—H13	0.9500
C4—C5	1.390 (2)	C14—H14	0.9500
C4—Cl1	1.7367 (15)	C15—H15C	0.9800
C5—C6	1.385 (2)	C15—H15A	0.9800
C5—H5	0.9500	C15—H15B	0.9800
C6—C7	1.393 (2)	C16—H16C	0.9800
C6—H6	0.9500	C16—H16A	0.9800
C7—C8	1.393 (2)	C16—H16B	0.9800
C7—H7	0.9500		
			
O1—C1—N1	123.81 (12)	C14—C9—C10	122.26 (13)
O1—C1—C3	121.86 (12)	C14—C9—N2	117.73 (12)
N1—C1—C3	114.33 (11)	C10—C9—N2	119.95 (12)
C1—N1—C2	128.06 (11)	C9—C10—C11	117.91 (13)
C1—N1—H1	116.0	C9—C10—C15	121.91 (13)
C2—N1—H1	116.0	C11—C10—C15	120.18 (13)
N2—C2—N1	116.79 (11)	C12—C11—C10	119.49 (13)
N2—C2—S1	124.85 (10)	C12—C11—C16	119.89 (13)
N1—C2—S1	118.37 (10)	C10—C11—C16	120.61 (13)
C2—N2—C9	123.85 (11)	C13—C12—C11	121.55 (13)
C2—N2—H2	118.1	C13—C12—H12	119.2
C9—N2—H2	118.1	C11—C12—H12	119.2
C8—C3—C4	119.39 (12)	C14—C13—C12	119.64 (13)
C8—C3—C1	119.55 (12)	C14—C13—H13	120.2
C4—C3—C1	120.99 (12)	C12—C13—H13	120.2
C5—C4—C3	121.20 (13)	C13—C14—C9	119.12 (13)
C5—C4—Cl1	119.12 (11)	C13—C14—H14	120.4
C3—C4—Cl1	119.67 (11)	C9—C14—H14	120.4
C6—C5—C4	118.81 (14)	C10—C15—H15C	109.5
C6—C5—H5	120.6	C10—C15—H15A	109.5
C4—C5—H5	120.6	H15C—C15—H15A	109.5
C5—C6—C7	120.82 (14)	C10—C15—H15B	109.5
C5—C6—H6	119.6	H15C—C15—H15B	109.5
C7—C6—H6	119.6	H15A—C15—H15B	109.5
C6—C7—C8	119.76 (14)	C11—C16—H16C	109.5
C6—C7—H7	120.1	C11—C16—H16A	109.5
C8—C7—H7	120.1	H16C—C16—H16A	109.5
C3—C8—C7	119.99 (14)	C11—C16—H16B	109.5
C3—C8—H8	120.0	H16C—C16—H16B	109.5
C7—C8—H8	120.0	H16A—C16—H16B	109.5
			
O1—C1—N1—C2	7.9 (2)	C1—C3—C8—C7	−177.24 (12)
C3—C1—N1—C2	−172.60 (12)	C6—C7—C8—C3	1.1 (2)
C1—N1—C2—N2	2.5 (2)	C2—N2—C9—C14	−64.20 (17)
C1—N1—C2—S1	−177.20 (11)	C2—N2—C9—C10	118.53 (15)
N1—C2—N2—C9	171.32 (12)	C14—C9—C10—C11	−0.39 (19)
S1—C2—N2—C9	−8.96 (19)	N2—C9—C10—C11	176.76 (11)
O1—C1—C3—C8	74.09 (18)	C14—C9—C10—C15	179.07 (12)
N1—C1—C3—C8	−105.46 (15)	N2—C9—C10—C15	−3.78 (19)
O1—C1—C3—C4	−102.77 (16)	C9—C10—C11—C12	1.59 (19)
N1—C1—C3—C4	77.68 (16)	C15—C10—C11—C12	−177.88 (12)
C8—C3—C4—C5	−1.0 (2)	C9—C10—C11—C16	−178.78 (12)
C1—C3—C4—C5	175.84 (12)	C15—C10—C11—C16	1.75 (19)
C8—C3—C4—Cl1	179.64 (10)	C10—C11—C12—C13	−1.4 (2)
C1—C3—C4—Cl1	−3.49 (17)	C16—C11—C12—C13	178.98 (13)
C3—C4—C5—C6	1.6 (2)	C11—C12—C13—C14	−0.1 (2)
Cl1—C4—C5—C6	−179.05 (11)	C12—C13—C14—C9	1.29 (19)
C4—C5—C6—C7	−0.9 (2)	C10—C9—C14—C13	−1.1 (2)
C5—C6—C7—C8	−0.5 (2)	N2—C9—C14—C13	−178.27 (11)
C4—C3—C8—C7	−0.3 (2)		

### Hydrogen-bond geometry (Å, º)

**Table:**

*D*—H···*A*	*D*—H	H···*A*	*D*···*A*	*D*—H···*A*
N1—H1···S1^i^	0.88	2.46	3.3104 (15)	164
N2—H2···O1	0.88	2.00	2.6866 (17)	134

Symmetry code: (i) −*x*, −*y*, −*z*+1.

## References

[bb1] Allen, F. H. (2002). *Acta Cryst.* B**58**, 380–388. 10.1107/s0108768102003890 12037359

[bb2] Altomare, A., Burla, M. C., Camalli, M., Cascarano, G. L., Giacovazzo, C., Guagliardi, A., Moliterni, A. G. G., Polidori, G. & Spagna, R. (1999). *J. Appl. Cryst.* **32**, 115–119.

[bb3] Johnson, C. K. (1976). *ORTEPII* Report ORNL-5138. Oak Ridge National Laboratory, Tennessee, USA.

[bb4] Kabuto, C., Akine, S., Nemoto, T. & Kwon, E. (2009). *J. Cryst. Soc. Jpn*, **51**, 218–224.

[bb5] Molecular Structure Corporation & Rigaku (2001). *CrystalClear* MSC, The Woodlands, Texas, USA, and Rigaku Corporation, Tokyo, Japan.

[bb6] Rauf, M. K, Ebihara, M., Badshah, A. & Imtiaz-ud-Din, (2012). *Acta Cryst.* E**68**, o119. 10.1107/S1600536811052780 PMC3254466 22259406

[bb7] Sheldrick, G. M. (2008). *Acta Cryst.* A**64**, 112–122. 10.1107/S0108767307043930 18156677

[bb8] Wakita, K. (2001). *Yadokari-XG* http://www.hat.hi-ho.ne.jp/k-wakita/yadokari

